# *Prevotella*-Based Bacterial Mixture Influences Gut Microbiota Composition in Weaned Piglets

**DOI:** 10.3390/microorganisms14020279

**Published:** 2026-01-25

**Authors:** Jitka Matiasovicova, Daniela Karasova, Alena Sebkova, Vladimir Babak, Ivan Rychlik

**Affiliations:** Veterinary Research Institute, 621 00 Brno, Czech Republic; matiasovicova@vri.cz (J.M.); karasova@vri.cz (D.K.); asebkova@vri.cz (A.S.); babak@vri.cz (V.B.)

**Keywords:** piglet, weaning, gut microbiota, *Bacteroides*, *Prevotella*, probiotic

## Abstract

Based on previous knowledge on changes in the gut microbiota of weaned piglets, a mixture of five different *Prevotella* species, *Anaerovibrio lipolyticus*, and *Mitsuokella multacida* (a *Prevotella* mixture) was tested as potentially novel type of probiotics for weaned Large White piglets of mixed sexes. The mixture was provided orally on the day of weaning to piglets in the experimental group, and the microbiota composition at weaning and one week later was determined by 16S rRNA sequencing in rectal swabs of 14 control and 27 experimental piglets. *Bacteroides* and *Escherichia* significantly decreased, and *Prevotella*, *Blautia*, or *Faecalibacterium* increased in the microbiota of both control and experimental piglets one week after weaning. Bacteria from the *Prevotella* mixture were detected in the gut microbiota of experimental piglets; however, the same bacteria of environmental origin were also recorded in control piglets. Despite this, early and uniform administration of the *Prevotella* mixture affected the composition of the gut microbiota of experimental piglets one week after weaning. The families Lactobacillaceae and Lachnospiraceae were more abundant in the gut microbiota of experimental piglets, while Pasteurellaceae, Coriobacteriaceae, Bacteroidales RF16 group, and Methanobacteriaceae were more abundant in control piglets. The *Prevotella*-based bacterial mixture thus may represent a novel approach to modify gut microbiota and consequently gut health in weaned piglets.

## 1. Introduction

Eukaryotic organisms are colonised by complex microbial populations, with the densest microbial communities found in the intestinal tract, where gut microbiota affects nutrient digestion and resorption. Consequently, the optimal structure of gut microbiota is projected into gross efficiency in farm animal production. The composition of gut microbiota changes during life. It develops with age or is affected by environmental conditions [1,2,3]. Gut microbiota composition is affected by diet, and differences in microbial communities in the intestinal tract can be recorded after antibiotic therapy [4,5]. The structure of gut microbiota can also be manipulated by administration of viable bacterial cultures, i.e., probiotics [6,7,8]. Concerning age, there are two critical time points in the development of gut microbiota in pigs and mammals in general—the first one is immediately after birth, when the nearly sterile intestinal tract becomes colonised [9], and the second one is after weaning when diet changes abruptly, and this is associated with adjustments in the gut microbiota composition [10,11].

In warm-blooded omnivorous vertebrates, gut microbiota usually consist of representatives of two major phyla, Bacteroidetes and Firmicutes, together with minority phyla, Actinobacteria and Proteobacteria [12]. In pigs and piglets, and within phylum Bacteroidetes, species from the genus *Bacteroides* are characteristic for nursed piglets, and these are replaced by species belonging to the related genus *Prevotella* after weaning [3,13,14,15,16,17,18,19,20]. A milk diet containing protein, sugars, and fats of pig origin therefore positively selects for *Bacteroides*, while an adult type of diet consisting of nutrients of plant origin supports the growth of *Prevotella* [10,21]. Similar enrichment for these genera has been described in human gut microbiota, with *Prevotella* being enriched in humans consuming a diet rich in vegetable and plant fibres, while *Bacteroides* dominates in humans consuming a diet rich in protein, sugars, and fat [22,23]. While a detailed understanding of substrate preferences by individual gut microbiota members will require additional studies, it is possible to adopt these principles and use them for the design of probiotics appropriate for a particular age category—*Bacteroides*-based probiotics for newborn piglets [24,25] and *Prevotella*-based probiotics for weaned piglets.

Probiotic treatment has been tested in weaned piglets mostly with representatives of the genera *Lactobacillus* and *Bacillus* [6,7,8,26]. Although positive outputs were reported, such protocols are not used in practise widely, likely due to their moderate effect on gut microbiota composition and host performance [27,28]. This is why alternative protocols mimicking the natural processes occurring in the gut microbiota of healthy piglets are tested as well [1,24,25]. A positive effect of experimentally provided *Prevotella copri* on the gut health of weaned piglets has been reported recently [29]; however, the presence of the used *Prevotella copri* strain in the gut microbiota of treated piglets was not determined in this study, which makes the meaning of all the recorded data uncertain.

In the current study, we followed the natural development of piglet gut microbiota after weaning and tested a mixture of *Prevotella* species in weaned piglets. Modification of the gut microbiota composition in weaned piglets is more challenging than in newborn piglets with a naive composition of gut microbiota. Weaned piglets at the time of any probiotic administration are 3–4 weeks of age, and for all this time, they are in contact with an adult sow, which acts as a source of adult-type microbiota. Rather moderate effects should therefore be expected. However, even in such cases, it might be useful to provide all piglets in a litter or herd with appropriate probiotics on the day of weaning, and position all of them at the same starting line. Such an intervention may decrease the occurrence of poorly colonised piglets in a litter which would otherwise act as reservoirs and seeders of undesired microbiota members. In this study, we therefore tested (i) whether it is possible to use novel bacterial species as alternatives to commonly used Lactobacilli, Enterococci, or Bifidobacteria [30], (ii) whether these species are able to colonise piglets after a single-dose administration on the day of weaning, and (iii) whether their administration affects the development of gut microbiota after weaning.

## 2. Materials and Methods

### 2.1. Ethics Statement

The handling of animals in this study was performed in accordance with current Czech legislation (Animal Protection and Welfare Act No. 246/1992 Coll. of the Government of the Czech Republic). The specific experiments were approved by the Ethics Committee of the Veterinary Research Institute, followed by the Committee for Animal Welfare of the Ministry of Agriculture of the Czech Republic on 3 March 2023 (permit number MZe 2406).

### 2.2. Bacterial Strains and Culture

Rectal swabs from 10 adult pigs were resuspended in 5 mL of PRAS (0.1 g magnesium sulphate heptahydrate, 0.2 g monobasic potassium phosphate, 0.2 g potassium chloride, 1.15 g dibasic sodium phosphate, 3.0 g sodium chloride, 1.0 g sodium thioglycolate, 0.5 g L-cysteine, and 1000 mL distilled water; final pH: 7.5 ± 0.2 at 25 °C) in an anaerobic chamber. The samples were serially diluted, plated on Wilkins–Chalgren anaerobe agar (Oxoid, Basingstoke, UK), and incubated in an anaerobic chamber under an atmosphere consisting of 10% CO_2_, 5% H_2_, and 85% N_2_ at 37 °C for 48 h. Approximately ten colonies of different morphology were selected from each agar plate, subcultured, and tested for growth under aerobic conditions at 37 °C for 48 h. Aerobically growing cultures were discarded, and isolates of strict anaerobes were stored at −70 °C in 1 mL aliquots of BHI medium (Oxoid, Basingstoke, UK) containing glycerol at 10% concentration. To assign the obtained isolates to bacterial species, DNA purified using a DNeasy Blood & Tissue Kit (Qiagen, Hilden, Germany) was used as a template in the PCR, amplifying over the whole 16S rRNA gene using forward TGAAGAGTTTGATCATGGCTCAG and reverse AGGAGGTGATCCAGCCGCA primers. The resulting PCR product was subjected to Sanger sequencing, with the forward primer using an external service (Eurofins, Prague, Czech Republic). The obtained sequences were BLAST-compared with the GenBank database (https://blast.ncbi.nlm.nih.gov/Blast.cgi, accessed on 20 January 2026), and selected isolates were subjected to whole-genome sequencing using an external service (Eurofins, Prague, Czech Republic). The whole genomic sequences were assembled using Unicycler and SPAdes [31,32] followed by annotation with RAST [33]. After this analysis, isolates of *Prevotellamassilia timonensis* An873, *Prevotella stercorea* An881, *Prevotella copri* An893, *Prevotella dentalis* An868, *Prevotella brevis* An923, *Anaerovibrio lipolyticus* An883, and *Mitsuokella multacida* An892 were selected for subsequent experiments. The mixture consisting of 5 representatives of the Prevotellaceae family was therefore enriched by an additional 2 representatives of the Veillonellaceae family, which are also characteristic for the microbiota of weaned piglets [11,15,16]. Strains of these taxa efficiently colonise an appropriate host [34] and increase resistance to *E. coli* and *Salmonella* infection in chickens [35,36]. Because of the dominance of *Prevotella* species, the mixture is called as a *Prevotella* mixture in the rest of this study.

### 2.3. Animal Experiments

There were two groups of mixed-sex piglets from a local farm consisting of 14 control and 27 experimental piglets of Large White breed. Control and experimental piglets were kept in different pens within the same animal house. Control piglets originated from a single litter, while piglets from other two litters were included in the experimental group. All the piglets were provided the same feed formulated to meet the nutritional recommendations for weaned piglets. The piglets were weaned on day 27 of life, when piglets in the experimental group were treated orally with a single dose of the *Prevotella* mixture. Piglets in the control group remained without any treatment throughout the whole experiment. Rectal swabs were collected from all piglets in both groups on the day of weaning, before piglets in the experimental group were treated with the *Prevotella* mixture, and one week after weaning. For technical reasons (failure in DNA purification from rectal swabs), only 24 samples from piglets treated with the *Prevotella* mixture were available after weaning. This means that altogether, 79 samples were collected and analysed for microbiota composition in this study—14 from control and 27 from experimental piglets on the day of weaning, and 14 from control and 24 from experimental piglets one week after weaning, i.e., on day 34 of life.

### 2.4. Production of the Prevotella Probiotic Mixture

To prepare probiotic mixtures for newborn piglets, all 7 strains were grown individually in BHI Vegan medium (HiMedia, Thane, Maharashtra, India) in an anaerobic cabinet at 37 °C for 48 h. When the culture was completed, bacterial counts were approx. 1 × 10^8^ CFU/mL. Equal volumes of individual cultures were mixed, pelleted by centrifugation, resuspended in a 20-times-lower volume of BHI medium supplemented with 10% glycerol, and stored at −70 °C. A 100 µL aliquot has been used for DNA purification and 16S rRNA gene sequencing to verify the mixture composition and exclude any contaminations. Prior to use, the strains were delivered to the farm on dry ice in 1 mL aliquots. The volume of 1 mL was thawed in 19 mL of sterile PBS, and each piglet was orally inoculated with 1 mL of the suspension containing approx. 1 × 10^8^ CFU of all bacteria on the day of weaning.

### 2.5. Microbiota Characterisation by Sequencing of the V3/V4 Variable Region of the 16S rRNA Gene

The rectal swab samples were homogenised for 1 min at 7000 RPM in a MagNALyzer (Roche, Basel, Switzerland) using zirconia silica beads (BioSpec Products, Bartlesville, OK, USA). Following homogenization, the DNA was extracted using a QIAamp DNA Stool Mini Kit according to the manufacturer’s instructions (Qiagen, Hilden, Germany), and the DNA concentration was determined spectrophotometrically. DNA samples diluted to 5 ng/mL were used as a template in a polymerase chain reaction (PCR) with forward primer 341F 5′-TCG TCG GCA GCG TCA GAT GTG TAT AAG AGA CAG-MID-GTC CTA CGG GNG GCW GCA G-3′ and reverse primer 805R 5′-GTC TCG TGG GCT CGG AGA TGT GTA TAA GAG ACA G-MID-GTG ACT ACH VGG GTA TCT AAT CC-3′ amplifying over the V3/V4 variable region of the 16S rRNA gene. The MIDs shown above represent different sequences 5, 6, 7, or 9 bp in length that were used to identify individual samples in post-sequencing analysis. The PCR was initiated with denaturation at 95 °C for 3 min, followed by 30 cycles of denaturation at 98 °C for 20 s, annealing at 60 °C for 20 s, and extension at 72 °C for 15 s, followed by final extension at 72 °C for 5 min. PCR amplification was performed using a HotStarTaq Plus Master Mix kit (Qiagen, Hilden, Germany), and the resulting PCR products were purified using AMPure beads (Beckman Coulter, Brea, CA, USA). In the next step, the concentration of PCR products was determined spectrophotometrically, the DNA was diluted to 100 ng/µL, and groups of PCR products with different MID sequences were indexed with indices using a Nextera XT Index Kit (Illumina, San Diego, CA, USA). Prior to sequencing, the concentration of differently indexed samples was determined using a KAPA Library Quantification Complete kit (Kapa Biosystems, Wilmington, MA, USA). All indexed samples were diluted to 4 ng/µL, and 20 pM phiX DNA was added to a final concentration of 5% (*v*/*v*). Sequencing was performed using a MiSeq Reagent Kit v3 and MiSeq apparatus (Illumina, San Diego, CA, USA). Analysis of sequencing data was performed with QIIME2 v2021.2.0 with default settings [37]. Raw sequence data were demultiplexed and quality filtered, and sequencing primers were removed using Je [38] and fastp [39]. The resulting sequences were denoised with DADA2 [40]. Taxonomy was assigned to ASVs using the q2-feature-classifier [41] and classify-sklearn naive Bayes taxonomy classifier against Silva 138 [42]. All the software tools were used with default settings.

### 2.6. Statistics

To identify key events in microbiota development in weaned piglets, and following *Prevotella* mixture administration, 4 different groups of piglets were defined. The first group was formed by control piglets sampled on the day of weaning. Since the piglets were weaned on day 27 of life, samples from these piglets were designated as Control27. Samples collected from the same piglets one week later were then designated as Control34. Piglets treated with the *Prevotella* mixture were designated as Probio and those sampled on the day of weaning were designated as Probio27. The last group of samples from piglets treated with the *Prevotella* mixture and sampled 7 days after weaning was designated as Probio34.

Identification of differently abundant taxa was performed by the Kruskal–Wallis test (R-project 4.5.1, package stats) followed by Dunn’s test for pairwise comparisons (R-project, package rstatix) and Linear Discriminant Analysis Effect Size (LEfSe; R-project 4.5.1, packages phyloseq and microbiomeMarker). The Kruskal–Wallis test was used to compare abundance at family, genus, and ASV level, while LEfSe was used for defining the most characteristic difference at all taxonomic levels. Only ASVs that were present in more than 15 piglets (out of 79 subjected to 16S rRNA sequencing) were subjected to statistical analysis. Such an inclusion threshold was used to focus on major taxa and major events. In other words, using this threshold, we focused on ASVs that were present in at least 20% of the analysed samples. Significant differences (*p* < 0.05) were recorded from comparisons between Control27 and Control34 groups, and Probio27 and Probio34 groups, to check for changes in microbiota composition before and after weaning. Comparison between Control34 and Probio34 groups was used to identify the effect of *Prevotella* mixture administration. Finally, we also checked for differences between Control27 and Probio27 piglets to exclude microbiota members differently abundant in both groups of piglets at the beginning of the experiment, prior to treatment with the *Prevotella* mixture. Statistical significance is not mentioned in the rest of this paper, but whenever a particular taxon is mentioned as more (or less) abundant in some of the compared groups, such a comparison reached statistical significance—comparative adjectives are never used without being supported by statistical significance.

## 3. Results

### 3.1. Microbiota Composition Before and After Weaning

**Figure 1:**
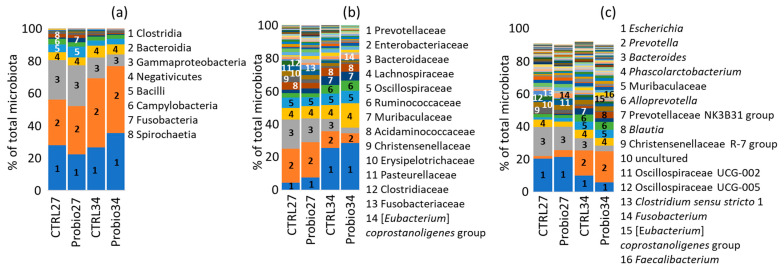
Composition of gut microbiota of 27- and 34-day-old piglets. (**a**) Microbiota composition at the class level. (**b**) Microbiota composition at the family level. (**c**) Microbiota composition at the genus level. The top 100 genera are included in panel c, so that the total sum does not reach 100%. Only the main taxa are identified in all panels. For the full dataset, see Table S1. CTRL27—control piglets sampled at weaning on day 27 of life; Probio27—piglets in the experimental group sampled at weaning on day 27 of life before treatment with the *Prevotella* mixture; CTRL34—control pigs sampled on day 34 of life; Probio34—pigs in the experimental group sampled on day 34 of life, one week after treatment with the *Prevotella* mixture.

The microbiota of piglets on days 27 and 34 of life, i.e., on the day of weaning and one week later, was dominated by representatives of Clostridia, Bacteroidia, and Gammaproteobacteria (Figure 1a). The most abundant families comprised Prevotellaceae, Enterobacteriaceae, Bacteroidaceae, Lachnospiraceae, Oscillospiraceae, Ruminococcaceae, Muribaculaceae, Acidaminococcaceae, Christensenellaceae, and Erysipelotrichaceae (Figure 1b). At the genus level, *Escherichia*, *Prevotella*, *Bacteroides*, *Phascolarctobacterium*, unclassified Muribaculaceae, *Alloprevotella*, Prevotellaceae NK3B31 group, *Blautia*, and Christensenellaceae R-7 group formed more than 50% of the total microbiota in piglets 27 or 34 days of age (Figure 1c).

### 3.2. Microbiota Development After Weaning

**Figure 2:**
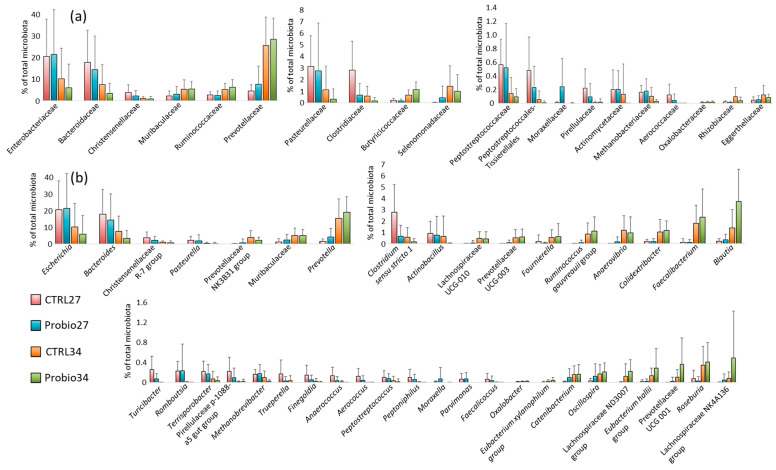
Microbiota development within the first week after weaning. Differently abundant taxa on the day of weaning and one week later, both in control and experimental piglets, at the family (**a**) and genus (**b**) level by the Kruskal–Wallis test (*p* < 0.05), i.e., all comparisons between CTRL27 and CTRL34, and also between Probio27 and Probio34, were found to be significant. Multiple graphs with different Y-axis scaling have been used to facilitate the readout of taxa with different abundances in the tested samples. CTRL27—control piglets sampled at weaning on day 27 of life; Probio27—piglets in the experimental group sampled at weaning on day 27 of life before treatment with the *Prevotella* mixture; CTRL34—control pigs sampled on day 34 of life; Probio34—pigs in the experimental group sampled on day 34 of life, one week after treatment with the *Prevotella* mixture.

Changes in microbiota composition at the time of weaning and one week later were analysed independently for control and experimental pigs, and then taxa with significantly different abundance both in control and probiotic-treated piglets were identified. At the family level, the abundance of Enterobacteriaceae, Bacteroidaceae, and Christensenellaceae significantly decreased, and that of Muribaculaceae, Ruminococcaceae, and Prevotellaceae significantly increased after weaning, both in control and experimental piglets (Figure 2a). Among the moderately abundant families, those that decreased within the first week after weaning comprised the families Pasteurellaceae, Clostridiaceae, Peptostreptococcaceae, Peptostreptococcales-Tissierellales, Moraxellaceae, Pirellulaceae, Actinomycetaceae, Methanobacteriaceae, and Aerococcaceae. On the other hand, the moderately represented families that increased in abundance after weaning included Butyricicoccaceae, Selenomonadaceae, Oxalobacteraceae, Rhizobiaceae, and Eggerthellaceae (Figure 2a).

At the genus level, *Escherichia*, *Bacteroides*, Christensenellaceae R-7 group, and *Pasteurella* decreased after weaning. On the other hand, *Prevotella*, Prevotellaceae NK3B31 group, and unclassified Muribaculaceae increased after weaning (Figure 2b). Out of the moderately abundant genera, those that decreased within the first week after weaning comprised *Clostridium sensu stricto* 1, *Actinobacillus*, *Turicibacter*, *Romboutsia*, *Terrisporobacter*, Pirellulaceae p-1088-a5 gut group, *Methanobrevibacter*, *Trueperella*, *Finegoldia*, *Anaerococcus*, *Aerococcus*, *Peptostreptococcus*, *Peptoniphilus*, *Moraxella*, *Parvimonas,* and *Faecalicoccus*. On the other hand, the moderately represented genera that significantly increased in abundance after weaning included *Blautia*, *Faecalibacterium*, *Colidextribacter*, *Anaerovibrio*, [*Ruminococcus*] *gauvreauii* group, *Fournierella*, Prevotellaceae UCG-003, Lachnospiraceae UCG-010, Lachnospiraceae NK4A136 group, *Roseburia*, Prevotellaceae UCG-001, [Eubacterium] hallii group, Lachnospiraceae ND3007 group, *Oscillospira*, *Catenibacterium*, [*Eubacterium*] *xylanophilum* group, and *Oxalobacter* (Figure 2b).

### 3.3. Differently Abundant Microbiota Members After Treatment with the Prevotella Mixture

**Figure 3:**
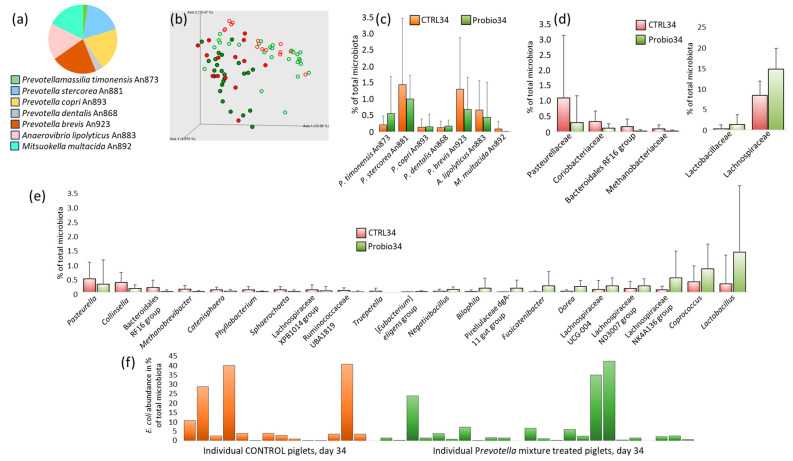
Differently abundant taxa in piglets treated with the *Prevotella* mixture. (**a**) Composition of the bacterial mixture used for piglet treatment. (**b**) PCoA clustering using unweighted Unifrac distances. Green symbols denote piglets treated with the *Prevotella* mixture, and red symbols denote control piglets. Open symbols denote piglets sampled on day 27 of life, i.e., on the day of weaning. Closed symbols denote piglets sampled on day 34 of life. (**c**) The abundance of bacteria present in the *Prevotella* mixture did not differ in the gut microbiota of control and experimental piglets. (**d**,**e**) Differently abundant taxa in control and experimental piglets at the family (**d**) and genus (**e**) level by the Kruskal–Wallis test (*p* < 0.05), i.e., all shown comparisons were found to be significant. *E. coli* formed more than 10% of the total microbiota in 4 out of 14 control piglets but in only 3 out of 24 experimental piglets one week after weaning (**f**). CTRL34—control pigs sampled on day 34 of life; Probio34—pigs in the experimental group sampled on day 34 of life, one week after treatment with the *Prevotella* mixture.

Next, we compared which taxa were influenced by the administration of the *Prevotella* mixture, first checking for the presence of strains from the *Prevotella* mixture in the inoculum and in the treated piglets. The inoculum contained all 7 strains and no other contaminants (Figure 3a). All used strains were also found in the microbiota of treated piglets one week after administration. *Prevotellamassilia timonensis* An873 forming 0.56 ± 1.14% of the total microbiota, *Prevotella stercorea* An881 (1.01 ± 0.72%), *Prevotella copri* An893 (0.15 ± 0.39%), *Prevotella dentalis* An868 (0.17 ± 0.19%), *Prevotella brevis* An923 (0.69 ± 0.99%), *Anaerovibrio lipolyticus* An883 (0.44 ± 1.07%), and *Mitsuokella multacida* An892 (0.004 ± 0.013%) were all detected in the microbiota of experimental piglets. However, the same ASVs were also present in the microbiota of control piglets. *Prevotellamassilia timonensis* formed 0.21 ± 0.27% of the total microbiota in control piglets, and *Prevotella stercorea* (1.45 ± 2.04%), *Prevotella copri* (0.14 ± 0.25%), *Prevotella dentalis* (0.12 ± 0.2%), Prevotella brevis (1.31 ± 1.58%), *Anaerovibrio lipolyticus* (0.67 ± 0.9%), and *Mitsuokella multacida* (0.09 ± 0.22%) were recorded in the microbiota of control piglets as well (Figure 3b). It was therefore impossible to decide on the origin of the strains from the *Prevotella* mixture in the microbiota of experimental piglets.

Despite this, the microbiota composition between control and experimental piglets one week after weaning was compared. PCoA clustering confirmed differences in microbiota composition before and after weaning, but indicated only a minor separation of control and experimental piglets after treatment with the *Prevotella* mixture (Figure 3c). Looking at taxa differentiating control and experimental piglets, the families Lactobacillaceae and Lachnospiraceae were more abundant in the gut microbiota of experimental piglets, while Pasteurellaceae, Coriobacteriaceae, Bacteroidales RF16 group, and Methanobacteriaceae were more abundant in control piglets one week after weaning (Figure 3d).

At the genus level, 21 genera were differently abundant in the microbiota of control and experimental piglets. Of these, 10 genera were more abundant in control piglets and 11 in the piglets treated with the *Prevotella* mixture. Those more abundant in control piglets included *Pasteurella*, *Collinsella*, Bacteroidales RF16 group, *Methanobrevibacter*, *Catenisphaera*, *Phyllobacterium*, *Sphaerochaeta*, Lachnospiraceae XPB1014, UBA1819 group, and *Trueperella*. On the other hand, the genera more abundant in experimental piglets included [*Eubacterium*] *eligens* group, *Negativibacillus*, *Bilophila*, Rikenellaceae dgA-11 gut group, *Fusicatenibacter*, *Dorea*, Lachnospiraceae UCG-004, Lachnospiraceae ND3007, Lachnospiraceae NK4A136, *Coprococcus*, and *Lactobacillus* (Figure 3e). Although *E. coli* was not differently abundant in the gut microbiota of control and experimental piglets one week after weaning, dominance of *E. coli* (forming more than 10% of the total microbiota) was more frequent in control piglets (in 4 out of 14 tested) in comparison to experimental piglets (in 3 out of 24 tested) (Figure 3f).

### 3.4. Taxa Characteristic for Individual Groups of Pigs

**Figure 4:**
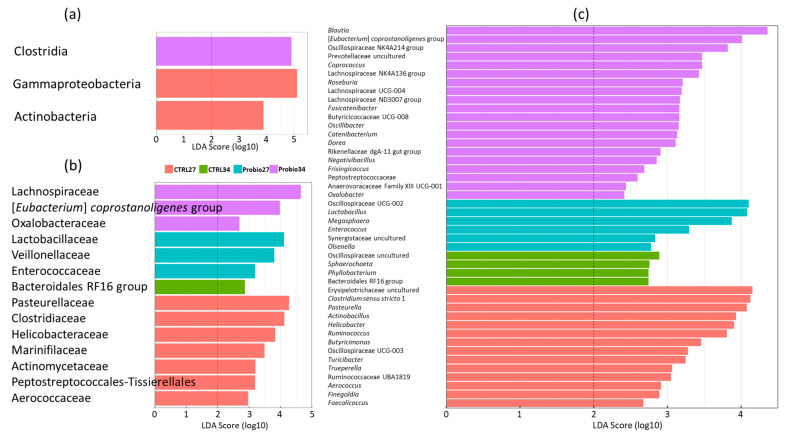
LefSe analysis of gut microbiota of control and *Prevotella*-mixture-treated piglets. LEfSe analysis identified the most discriminatory taxa at the class- (**a**), family- (**b**), and genus-level (**c**).

Finally, we identified the most characteristic taxa for each group using LEfSe analysis. Enrichment of class Clostridia was characteristic for piglets treated with the *Prevotella* mixture one week after weaning. On the other hand, Gammaproteobacteria and Actinobacteria were characteristic for control piglets on the day of weaning (Figure 4a). Fourteen families were characteristic for this particular group of piglets. Pasteurellaceae, Clostridiaceae, Helicobacteriaceae, Marinifilaceae, Actinomycetaceae, Peptostreptococcales-Tissierellales, and Aerococcaceae were characteristic for control piglets at weaning. Lactobacillaceae, Veillonellaceae, and Enterococcaceae were characteristic for the piglets at weaning that were later treated with the *Prevotella* mixture. Bacteroidales RF16 group was typical for the gut microbiota of control piglets one week after weaning, and Lachnospiraceae, *Eubacterium coprostanoligenes* group, and Oxalobacteriaceae were characteristic for the gut microbiota of experimental piglets one week after treatment with the *Prevotella* mixture (Figure 4b).

Altogether, 44 different genera were characteristic for one of the groups of piglets in this experiment, with the highest number of characteristic genera identified in experimental piglets one week after treatment with the *Prevotella* mixture (*n* = 20). Of the most characteristic, uncultured Erysipelotrichaceae, *Clostridium sensu stricto* I, *Pasteurella*, *Actinobacillus*, or *Helicobacter* were typical for the gut microbiota of control piglets at the time of weaning. *Lactobacillus*, *Megasphaera*, and *Enterococcus* were characteristic for experimental piglets on the day of weaning. Uncultured Oscilospiraceae, *Sphaerochaeta*, *Phylobacterium*, and Bacteroidales RF16 group were typical for the microbiota of control piglets one week after weaning, and *Blautia*, *Eubacterium coprostanoligenes*, *Coprococcus*, *Roseburia*, *Fusicatenibacter*, *Oscillibacter, Dorea*, and five poorly characterised genera from the families Lachnospiraceae, Oscillospiraceae, and Butyricicoccaceae were typical for experimental piglets one week after treatment with the *Prevotella* mixture (Figure 4c).

## 4. Discussion

Postweaning diarrhoea is a common disorder of the gastrointestinal tract in weaned piglets [27,43]. It is influenced by the stress caused by the separation of piglets from sows, the change in diet, and the accompanying changes in gut microbiota [10,21,44]. Changes in gut microbiota in weaned piglets have been described, and besides a general increase in diversity and decrease in *E. coli*, the replacement of taxonomically related Bacteroidaceae with Prevotellaceae is one of the repeatedly reported patterns [11,17,18,19], which we also confirmed in this study. In addition, there are reports on the positive correlation of *Prevotella* and body weight increases in weaned piglets [18]. Despite this, *Prevotella* has been tested as a probiotic in weaned piglets only once [29], while species such as *Bacillus* sp. or *Clostridium butyricum*, not mentioned as characteristic for weaned piglet microbiota, are tested together with Lactobacilli [6,7,8,27,45,46,47,48,49]. The fact that *Prevotella* is common in weaned piglets [20] may also argue against the use of these bacteria, since it might be difficult to compete with *Prevotella* of natural origin. Piglets at the time of weaning are in intimate contact with sows for 3–4 weeks, which represents a time long enough for the transfer of sow gut microbiota to nursed piglets. The administration of probiotic strains to weaned piglets can therefore be expected to be more challenging than the probiotic treatment of newborn piglets [24], which we confirmed in this study by finding the same *Prevotella* strains in the gut microbiota of both control and experimental piglets. It is possible that if sampling earlier, the differences in abundance of the strains from the *Prevotella* mixture could have been found to be significant. Similarly, if sampling more than one week after the administration of the *Prevotella* mixture, long-term benefits could have been recorded. Our observations also argue for a need to control the presence of used probiotic strains in treated animals. Reporting differences in multiple physiological parameters between control and experimental groups without knowing whether the used strains were present or absent from the gut microbiota of the experimental group, or were present both in control and experimental pigs, as in our case, may lead to misleading conclusions.

Despite the presence of strains from the *Prevotella* mixture in both control and experimental piglets, there were differences in other gut microbiota members of the control and experimental piglets one week after weaning. The microbiota of experimental piglets was enriched for representatives of the family Lachnospiraceae (*Blautia*, *Dorea*, or *Coprococcus*, or Lachnospiraceae of UCG-004, ND3007, or NK4A136 groups), which are taxa associated with the correct development of the gut microbiota of pigs after weaning [11,15,16,50]. This indicates that early and uniform administration of the *Prevotella* mixture to all piglets in the experimental group had a beneficial effect. We therefore speculate that *Prevotella*-treated piglets were generally healthier, with accompanying exploratory behaviour that resulted in more likely exposure to spore-forming bacteria present in the environment. Indeed, Lachnospiraceae comprise spore-forming species [51] surviving in the external environment in a form of spores, from which they infect susceptible hosts [52]. In chickens, these species are the first ones to colonise newly hatched chicks due to their ubiquitous presence in the environment [53,54], and these taxa are less abundant in autistic children in comparison to healthy controls [55], due to their less active behaviour.

Treatment with the *Prevotella* mixture caused significant reduction in methanogenic *Methanobrevibacter*. Pigs are not considered as a main source of methane, and the low abundance of *Methanobrevibacter* in piglet microbiota (around 0.15% of the total microbiota) confirms this. *Methanobrevibacter* decreased in both control and experimental piglets after weaning, but its decrease in experimental piglets was more extensive, resulting in a significant difference between control and experimental piglets one week after weaning, which is worthy of further investigation.

Similarly to *Methanobrevibacter*, *E. coli* also decreased in both control and experimental piglets one week after weaning, similar to previous reports [14,17,19]. Unlike *Methanobrevibacter*, the abundance of *E. coli* did not differ significantly in the microbiota of control and experimental piglets one week after weaning. However, there was a high proportion of weaned piglets with an *E. coli* abundance higher than 10% among the control piglets. Such piglets could act as a reservoir of *E. coli* for the remaining piglets in the herd, and may increase infectious pressure above the threshold, resulting in *E. coli*-caused post-weaning diarrhoea.

## 5. Conclusions

This study is limited by its focus on gut microbiota only, i.e., with no association of the *Prevotella* mixture treatment with host-response or production parameters. Despite this, we have shown that weaned piglets can be treated with a mixture consisting of *Prevotella*, *Anaerovibrio*, and *Mitsuokella* without any adverse effects. Although the same taxa were also found in the microbiota of control piglets, rapid and uniform administration of this mixture immediately after weaning to all piglets in a newly formed herd resulted in moderate changes in gut microbiota composition characteristic for healthy piglets. Representatives of *Prevotella* can therefore be tested as novel types of probiotics for weaned piglets. Their administration to weaned piglets can be combined with *Bacteroides*-based probiotics for newborn piglets [24], thus driving the correct development of gut microbiota in piglets from the moment of birth through weaning to the fattening period. Moreover, it might be of interest to combine the administration of bacterial strains with probiotic potential with microbiota members belonging to completely different kingdoms. Different *Prevotella* species can be combined with pig-adapted yeasts and/or phages specific for enterotoxigenic *E. coli* to achieve synergistic effects and further increase the resistance of weaned piglets to enteric diseases [56,57,58,59].

## Data Availability

The data presented in this study are openly available in GenBank at https://www.ncbi.nlm.nih.gov/bioproject/?term=PRJNA1357106, accession number [RJNA1357106] since 5 November 2025.

## Supplementary Materials

The following supporting information can be downloaded at: https://www.mdpi.com/article/10.3390/microorganisms14020279/s1, Table S1: List of ASVs recorded in this study.
